# Advancements and Clinical Applications Prospects of Epigenetic Biomarkers in Liquid Biopsy for Oral Squamous Cell Carcinoma

**DOI:** 10.3390/cimb48070680

**Published:** 2026-07-01

**Authors:** Yuan Li, Yao Liu, Yuyi Cong, Juan Liu, Wen Pan, Xiaobing Guan, Jiaqi Wang

**Affiliations:** 1 Department of Oral Medicine, School of Stomatology, Capital Medical University, Beijing 100070, China; 2 Department of Oral and Maxillofacial Surgery, School of Stomatology, Capital Medical University, Beijing 100070, China

**Keywords:** oral squamous cell carcinoma, oral potentially malignant disorders, potential epigenetic biomarkers, saliva, oral liquid biopsy, DNA methylation, non-coding RNA, cell-free DNA

## Abstract

Oral squamous-cell carcinoma (OSCC) is a prevalent malignancy of the head and neck region. A delay in the diagnosis of OSCC often results in a high metastatic tendency, which is the main reason for the high patient mortality. Dynamic monitoring and management of the onset and progression of OSCC are critical for improving patient survival rates. Liquid biopsy technology—characterized by its non-invasive nature, procedural convenience, and capacity for longitudinal monitoring—is a promising adjunct to histopathological examination for the early diagnosis of OSCC. Epigenetic alterations, characterized by reversibility and long-term stability in physiological fluids, are critical enablers of liquid biopsy and its clinical utility. Advances in detection technologies, including quantitative polymerase chain reaction (qPCR), digital droplet PCR (ddPCR), next-generation sequencing (NGS), and electrochemical biosensors, have significantly facilitated the research and clinical translation of epigenetic biomarkers in oral liquid biopsies. However, translating epigenetic biomarkers from research discovery to clinical practice for OSCC remains hindered by several critical challenges: the scarcity of large-scale, rigorously designed cohort studies, limited multicenter validation, inconsistent preprocessing protocols, and a lack of harmonized analytical platforms. Finally, we propose a conceptual framework to outline potential clinical application models for these biomarkers.

## 1. Introduction

Oral cancer is a significant global public health challenge, with OSCC accounting for approximately 90% of cases [1]. The majority of OSCC cases arise from oral potentially malignant disorders (OPMDs) through a multistep, protracted, and biologically complex carcinogenic process. Despite advances in oncology, the five-year overall survival rate for patients with advanced-stage OSCC remains below 50% [2,3]. This persistently high mor-tality is largely attributable to distant metastasis, which often arises as a consequence of delayed diagnosis [4,5]. Moreover, peritumoral tissues that appear normal clinically may exhibit molecular alterations indicative of early neoplastic or microenvironmental chang-es. Therefore, patients with OSCC should undergo longitudinal, dynamic surveillance using molecular biomarker profiling. Liquid biopsy is a pivotal diagnostic and monitor-ing tool in this process.

Currently, histopathological grading of oral mucosal epithelial dysplasia is the gold standard for diagnosing OSCC. Nevertheless, this approach has several limitations: the absence of objective, quantitative biomarkers, suboptimal inter-observer reproducibility, substantial subjectivity, and inter-pathologist variability. These shortcomings may compromise clinical decision-making [6,7,8,9,10,11]. Moreover, it is an invasive procedure that yields only limited tissue specimens, thereby limiting longitudinal, real-time monitoring of molecular dynamics during OSCC treatment. Consequently, reliance solely on histopathology precludes a dynamic assessment of disease progression and therapeutic response [12,13]. Although adjunctive techniques, including exfoliative cytology and autofluorescence imaging, have facilitated a shift toward minimally invasive diagnostic paradigms, no standardized, clinically validated molecular markers are currently available for early detection, lesion stratification, risk prediction, and treatment response monitoring [14]. Thus, novel, minimally invasive, sensitive, and clinically actionable diagnostic tools and biomarkers are a critical priority to optimize the precision diagnosis and management of OSCC. Advances in molecular biology, particularly high-throughput sequencing and mass spectrometry, have catalyzed the emergence of liquid biopsy, a non-invasive strategy for analyzing tumor-derived analytes in readily accessible biofluids. The liquid biopsy strategy overcomes the spatiotemporal constraints inherent to conventional tissue biopsies and enables serial, real-time molecular profiling of OSCC [15], with considerable promise in precision oncology. However, early liquid biopsy methodologies predominantly focused on detecting genetic mutations, often relying on a single, highly specific biomarker. This approach is inherently susceptible to analytical variability, particularly in the presence of intratumoral heterogeneity and low-abundance analytes, thereby compromising the accurate determination of tissue of origin. In this regard, epigenetic alterations, characterized by stability, cancer specificity, and early occurrence in carcinogenesis, have emerged as compelling candidates for next-generation liquid biopsy biomarkers.

**Figure 1:**
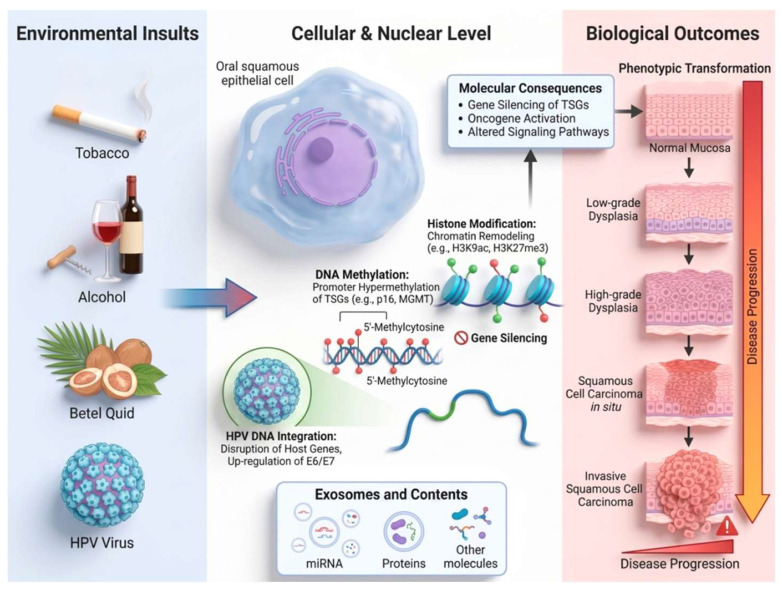
Epigenetic Mechanisms Underlying the Pathogenesis and Progression of Oral Squamous cell Carcinoma. The images were made with the assistance of NanoBanana 2.

The pathogenesis of OSCC is synergistically driven by environmental exposures, including tobacco smoking, alcohol consumption, and betel quid chewing, as well as genetic susceptibility factors such as high-risk human papillomavirus (HPV) infection. These factors modulate gene expression by altering epigenetic biomarks on chromatin, thereby reprogramming transcriptional regulation, effectively modifying the cellular “instruction manual,” and promoting malignant transformation of normal cells. Consequently, epigenetic mechanisms serve as a critical molecular interface linking environmental exposures to the pathogenesis of OSCC. Unlike irreversible genetic mutations, epigenetic alterations remain stable in biofluids and pharmacologically reversible, rendering them promising candidates for non-invasive, independent diagnostic biomarkers. Epigenetic biomarkers significantly expand the analytical scope of the liquid biopsy technology, facilitating a paradigm shift from phenotypic association toward mechanistic insights into OSCC biology [16,17,18] (Figure 1).

Among biofluids, saliva exhibits particular promise as a proximal diagnostic medium for OSCC. Due to its direct anatomical and physiological contact with the oral mucosa, saliva readily captures tumor-derived molecular analytes, including microRNAs, exosomes, cell-free tumor DNA, and circulating tumor cells. A saliva sample has high biological relevance and spatial specificity, and its non-invasive collection, cost effectiveness, and robust target enrichment make it an ideal specimen for OSCC-focused liquid biopsy. Critically, integrating salivary sampling with epigenetic profiling enables ultrasensitive detection of molecular residual disease, thereby supporting dynamic risk stratification, early recurrence monitoring, and longitudinal assessment of treatment response beyond conventional imaging modalities [19]. Despite this potential, clinical validation of salivary epigenetic biomarkers remains limited. Few studies have rigorously evaluated their diagnostic accuracy, prognostic utility, or feasibility for real-world implementation. 

In the future, comprehensive clinical management strategies integrating molecular biomarkers from both tissue and liquid biopsies will enable stage-adapted, precision-driven patient care. This approach enables high-risk population stratification, early detection of malignant transformation, real-time monitoring of therapeutic responses, and accurate prediction of recurrence risk. Collectively, these capabilities reduce disease burden, improve cure rates, and enhance patient quality of life.

## 2. Materials and Methods

This narrative review synthesizes evidence from a systematic literature search conducted in PubMed and Web of Science from January 2010 to March 2026. Search terms encompassed “epigenetics,” “liquid biopsy,” “oral squamous cell carcinoma (OSCC),” “oral epigenetic biomarkers,” and “oral fluid biopsy biomarkers,” combined using Boolean operators (AND, OR) to maximize retrieval precision and comprehensiveness. Inclusion criteria: (1) clinical studies or scholarly reviews addressing the pathogenesis, progression, or early detection of OSCC; (2) study designs limited to randomized controlled trials, prospective cohort studies, case–control studies, or high-quality systematic reviews; (3) in cases of insufficient data for the validation of epigenetic and liquid biopsy biomarkers in OSCC, evidence from studies on head and neck squamous cell carcinoma (HNSCC) was incorporated to support the analysis; (4) incorporation of select data published before 2010 to provide historical context on the evolution of epigenetic and liquid biopsy methodologies in oral oncology. Exclusion criteria: (1) publications with no full-text availability (e.g., conference abstracts only); (2) duplicate entries in databases. Although formal risk-of-bias assessment tools were not applied, this review deliberately prioritizes higher-level evidence (e.g., meta-analyses and multicenter cohort studies) and explicitly acknowledges limitations in biomarker validation where applicable.

## 3. The Development and Challenges of Liquid Biopsy

As a non-invasive detection technology, liquid biopsy captures tumor-derived materials from blood, saliva, urine, and other body fluids, including circulating tumor DNA (ctDNA) [20], cell-free DNA/RNA (cfDNA/RNA), circulating tumor cells (CTCs), exosomes, and other materials. This approach enables comprehensive profiling of tumor genomic and molecular characteristics [21,22], facilitating early detection and dynamic monitoring of malignancies. The development of oral liquid biopsy will be a gradual evolution from initial exploration to multi-omics integration, and from technical breakthroughs to clinical applications, driven by technological innovation and clinical needs.

Before 1980, research predominantly focused on protein-based biomarkers, including carcinoembryonic antigen and squamous cell carcinoma antigen, as well as on metabolite classes such as amino acids and lipid derivatives in biofluids [23,24]. No biomarker has been identified that is both specific and exclusive to oral squamous cell carcinoma. The objective is to enable simultaneous detection of multiple tumors. These biomarkers offer well-established detection methodologies and cost effectiveness. Protein-based biomarkers often exhibit weak signal intensity in early disease stages, limited specificity, and susceptibility to confounding effects from benign conditions such as inflammation and infection [25]. Metabolite profiles are highly sensitive to extrinsic variables, including dietary intake and pharmacological interventions, and exhibit poor analytical stability. At present, with the development of advanced proteomics technologies, such as glycoprotein and glycan modification detection, the Olink adjacency ligation method, and single-molecule array (SIMOA), the sensitivity of protein markers has been expanded [26,27].

In the 1990s, pivotal methodological advances transformed liquid biopsy from an observational curiosity into a scientifically grounded approach. Using PCR, investigators successfully detected KRAS mutations in plasma-derived cell-free DNA (cfDNA) from patients with pancreatic cancer. This finding paralleled the detection of homologous tumor tissues, validating the feasibility of non-invasive genotyping via circulating nucleic acids. Concurrently, CTCs were reliably isolated and correlated with tumor stage and burden, signifying a paradigm shift from single-analyte detection to multimodal, biologically integrated analysis [28,29,30]. Currently, the clinical utility of circulating cfDNA and CTCs in body fluid samples from OSCC patients remains unvalidated, warranting additional rigorous clinical and analytical validation studies.

From 2000 to 2010, extensive clinical studies substantiated the prognostic and dynamic monitoring value of liquid biopsy biomarkers. Several studies have shown a significant correlation between CTC levels and survival outcomes in OSCC and other malignancies, including breast, lung, prostate, and ovarian cancers, highlighting the potential utility of CTCs for early detection of OSCC and longitudinal therapeutic monitoring. Furthermore, Partridge et al. [31] prospectively assessed CTC burden in peripheral blood and bone marrow samples from 40 patients with OSCC detected preoperatively and during surgical intervention. According to their findings, detectable CTCs were independently associated with an elevated risk of locoregional recurrence and distant metastasis. Collectively, these findings confirmed that liquid biopsy captures static genomic features of tumors and their real-time evolutionary dynamics, providing a rigorous evidence base for clinical integration.

**Figure 2:**
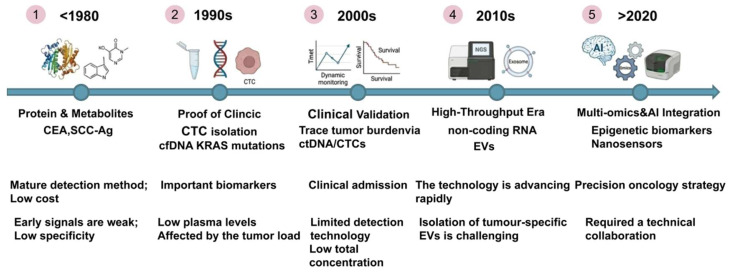
Development of liquid biopsy.

Between 2010 and 2020, the shift from traditional PCR-based methods to high-throughput sequencing catalyzed rapid growth in studies of tumor-derived liquid biopsy analytes such as cfDNA, cfRNA, and exosomes. Liquid biopsy is rapidly being adopted in routine oncology practice. For instance, plasma-based ctDNA testing for EGFR mutation detection has been approved by the U.S. FDA and is used as a companion diagnostic tool in non-small-cell lung cancer. Regulatory approvals and incorporation into evidence-based clinical guidelines reflect its maturation as a validated diagnostic tool. Since 2020, the field of oral liquid biopsy has entered a new phase characterized by the integration of epigenetic biomarkers and collaborative multi-omics approaches. Epigenetic biomarkers have promoted the evolution of OSCC liquid biopsy from “passive detection” to “active early-warning” approaches. Longitudinal monitoring of dynamic changes in *HOXA5* methylation in the saliva can predict the risk of OSCC in patients with oral leukoplakia (OLK). Aberrant expression of m^6^A modification-related molecules detectable in body fluids can effectively discriminate OSCC patients from healthy individuals and provide crucial guidance for targeted therapies [32,33,34]. Cell-free DNA (cfDNA) carries sequence information and multidimensional epigenetic features, including fragmentation patterns, end-motifs, nucleosome positioning, and methylation status. Integrating multidimensional epigenetic features of cfDNA yields a more comprehensive molecular map of tumor cells, enabling further expansion of liquid biopsy applications, improved characterization of tumor biology, treatment response, and drug resistance mechanisms, and ultimately enhanced clinical utility [35,36,37,38]. The core value of this generation of detection tools lies in their ability to provide comprehensive coverage across the entire continuum: from early screening and risk stratification to therapeutic efficacy monitoring. In the future, these tools hold significant potential as cornerstones for the precision diagnosis and treatment of OSCC. Liquid biopsy is currently recognized as a critical complementary tool to tissue biopsy in precision oncology. In OSCC, this evolution has shifted the research focus from conventional blood-based markers to locally accessible biofluids (particularly saliva) and to more stable, early-detectable epigenetic analytes, including DNA methylation signatures and non-coding RNAs (ncRNAs). However, most of these biomarkers are still in the exploratory phase, and no clinically validated biomarkers for OSCC have been established to date (Figure 2).

## 4. Potential Liquid Epigenetic Biomarkers

### 4.1. The Role of Epigenetic Mechanisms in OSCC

Epigenetics refers to heritable and reversible modifications in gene expression or cellular phenotypes that occur independently of changes in the underlying DNA nucleotide sequence. Key epigenetic mechanisms include DNA methylation [39], histone post-translational modifications [40], RNA modifications [41], chromatin remodeling, and regulation by ncRNAs [42]. Epigenetic regulatory proteins are commonly classified as “writers,” “erasers,” “readers,” and “remodelers” and are recruited to specific genomic loci [43,44,45,46], as they dynamically modulate chromatin structure and function in response to physiological or pathological stimuli. The ongoing identification of novel epigenetic regulators has increasingly underscored the functional reversibility of epigenetic marks. Consequently, targeted modulation of epigenetic enzymatic activity holds significant promise for developing novel epigenetically informed diagnostic and therapeutic strategies [47]. Importantly, disease-associated epigenetic alterations are not confined to tumor tissue; they are also stably detectable in accessible biofluids such as saliva and blood, thereby offering a compelling foundation for the development of non-invasive or minimally invasive biomarkers with high sensitivity and specificity.

### 4.2. The Advantages and Value of Epigenetics in Liquid Biopsy of OSCC

Environmental factors, including tobacco smoking, alcohol consumption, betel quid chewing, and HPV infection, interact with genetic susceptibility to drive the occurrence of OSCC [48]. Epigenetic modifications serve as a critical mechanistic link between environmental exposures and dysregulated gene expression in OSCC [49]. Compared with genomic mutations, epigenetic alterations typically arise earlier during carcinogenesis, which exhibit high reversibility and demonstrate superior stability in biofluids. Notably, these modifications can be robustly detected in clinically accessible specimens such as saliva and plasma, thereby enriching the biomarker repertoire for liquid biopsy development and advancing the precision and clinical utility of non-invasive OSCC diagnosis. Key advantages of epigenetic markers in OSCC liquid biopsy include: (1) early detection potential: epigenetic aberrations (e.g., aberrant DNA methylation or dysregulated ncRNAs) frequently emerge in premalignant conditions such as OLK and other OPMDs; (2) analytical stability: DNA methylation patterns and certain ncRNAs remain relatively resistant to degradation in biofluids, ensuring reproducible assay performance; (3) tumor specificity: the epigenetic landscape of OSCC cells is highly distinct from that of adjacent normal tissue, minimizing biological background interference; and (4) functional relevance: epigenetic alterations directly reflect pathogenic molecular processes, rendering them not only valuable diagnostic biomarkers but also promising therapeutic targets.

#### 4.2.1. Biomarkers of DNA Methylation

DNA methylation involves the covalent addition of methyl groups to the cytosine ring of CpG islands under DNA methyltransferase (DNMT) catalysis, thereby preventing transcription and inhibiting gene expression. However, DNA demethylase can reverse this process and restore gene expression activity [50]. As one of the earliest epigenetic mechanisms discovered in tumorigenesis [51], DNA methylation remodeling is a key early driver of OSCC progression. Tobacco exposure, a significant risk factor for OSCC, leads to genome-wide DNA hypomethylation [52]. The genome-wide OED-associated methylation pattern is a key predictor of OSCC risk. This genome-wide loss of methylation may induce chromosomal instability, increase the gene mutation burden, and drive tumorigenesis. On the other hand, hypomethylation of specific gene regions can also activate protective transcriptional programs, promote benign cell differentiation, and inhibit malignant transformation, thereby inhibiting tumorigenesis. Abnormal methylation in OSCC is characterized by the synergistic coexistence of “global hypomethylation” and “local hypermethylation.” This dynamic imbalance plays an important role in the development of OSCC. Therefore, analyzing differential methylation patterns of specific genes can serve as molecular markers for OSCC diagnosis. Meanwhile, monitoring the dynamic methylation profile of the whole genome may help predict the risk of carcinogenesis in high-risk groups.

In OSCC, hypomethylation of the promoter regions of specific genes can relieve transcriptional repression, leading to abnormal gene activation, disrupting cell cycle regulation and apoptotic homeostasis, and driving malignant transformation. These changes usually occur in proto-oncogenes.

Hypermethylation of specific gene promoters suppresses transcription via epigenetic mechanisms, thereby compromising critical cellular functions, including DNA damage response, cell cycle regulation, apoptosis induction, and tumor-suppressive defense pathways. Consequently, this epigenetic aberration induces uncontrolled cell proliferation and facilitates malignant tumor progression. Classical tumor suppressor genes frequently silenced epigenetically in OSCC include: DNA damage repair-related genes (*MGMT* and *DAPK*), cell cycle regulators (*p16/CDKN2A*), cell adhesion and metastasis inhibitors (*CDH1* and *TIMP3*), signaling pathway inhibitors (*APC* and *RASSF1A*), and differentiation and immune regulators (*TGM3* and *HOXA5*). The hypermethylation spectrum of the above genes drives the malignant progression of OSCC and has potential as a high-value diagnostic biomarker due to its stability and tissue specificity; however, verification has not yet been conducted in salivary samples. Table 1 summarizes the key characteristics of these methylation biomarkers. Although epigenetic regulatory factors exhibit considerable promise as OSCC biomarkers in preclinical research, current evidence largely relies on in vitro models and small-scale, single-center cohort studies. Furthermore, their clinical translatability has not yet been validated in readily accessible biofluids, such as peripheral blood or saliva, limiting both generalizability and statistical power. Rigorously designed, large-scale, multicenter, longitudinal clinical trials are essential to robustly establish their diagnostic and prognostic utility. Looking ahead, integrating genome-wide DNA methylation profiling with functionally informed, target-specific epigenetic interventions has the potential to advance precision oncology strategies for OSCC.

#### 4.2.2. Biomarkers of RNA Modification

RNA methylation is modified by methyltransferases (writers), while demethylases (erasers) are responsible for its reversible erasure. Methyltransferases precisely regulate RNA metabolism by recognizing conserved sequences and local RNA secondary structures, depositing methyl groups at specific sites, coordinating with the RNA polymerase II transcription elongation complex, or forming functional interaction networks with RNA-binding proteins [110]. The major RNA modifications include 7-methylguanosine (m^7^G), 5-methylcytosine (m^5^C), N^1^-methyladenosine (m^1^A), and N^6^-methyladenosine (m^6^A) [111].

N^6^-methyladenosine (m^6^A) modification is the most prevalent post-transcriptional RNA methylation in eukaryotic cells [112]. Evidence indicates that m^6^A levels increase significantly in hyperproliferative oral mucosa and OSCC epithelial tissues compared with normal oral epithelium, implicating m^6^A dysregulation in the pathogenesis of oral epithelial dysplasia (OED) [113]. Recent studies further demonstrate its functional roles in circular RNAs (circRNAs) and long non-coding RNAs (lncRNAs), thereby offering promising avenues for the diagnosis of and precision therapeutic interventions for OSCC. However, these findings are predominantly preclinical, and robust clinical validation, particularly using well-annotated human tissue cohorts, remains lacking. Key m^6^A regulatory factors, including writers (METTL3, METTL14, METTL5), erasers (ALKBH5), readers (YTHDF1-3, YTHDC1-2), and auxiliary proteins (RBMX, NSUN2), exert oncogenic or tumor-suppressive effects in OSCC through context-dependent modulation of m^6^A deposition. Aberrant expression of these factors correlates strongly with clinicopathological features and patient outcomes, positioning them as potential diagnostic and prognostic biomarkers. Nevertheless, the absence of validation in accessible biofluids (e.g., saliva and plasma) [114] remains a significant barrier to clinical application.

In contrast, the functional relevance of m^5^C modification in OSCC remains incompletely characterized. A recent integrative analysis of genomic and clinical data from 558 OSCC patients classified tumors into two distinct m^5^C-modified subtypes based on the expression profiles of 16 canonical m^5^C regulators, with marked differences in tumor immune microenvironment (TIME) composition, immune infiltration patterns, and clinical prognosis. Weighted gene co-expression network analysis (WGCNA) helped identify m^5^C-associated gene modules, enabling the development of a quantitative m^5^C scoring system. Patients with high m^5^C scores exhibited significantly elevated immune, stromal, and ESTIMATE scores, reduced tumor purity, attenuated anti-tumor immune activity, increased tumor mutational burden (TMB), markedly inferior overall survival (OS) and progression-free survival, and higher recurrence rates. Collectively, these findings suggest that m^5^C modification patterns shape the TIME and influence OSCC aggressiveness, with the m^5^C score holding promise as a novel, quantitative biomarker for stratifying risk, predicting prognosis, and monitoring malignant progression [115].

To date, the functional implications of m^7^G RNA modifications in OSCC remain incompletely characterized. The METTL1-catalyzed m^7^G RNA modification promotes OSCC cell proliferation, and NEK1 has been validated as a functionally relevant downstream effector. Collectively, these findings make METTL1 a promising prognostic biomarker and a rational therapeutic target in OSCC, underscoring the need for further mechanistic investigation and rigorous evaluation of its clinical utility.

#### 4.2.3. Biomarkers of Histone Modifications

Histones are highly basic proteins rich in lysine and arginine residues, around which DNA wraps to form nucleosomes—the basic chromatin unit. Histone modification refers to the chemical modification in specific amino acid residues in the N-terminal tail of histones. These modifications remodel the higher-order structure of chromatin by altering histone charge states or recruiting effector proteins, thereby regulating DNA accessibility and activating or repressing gene transcription. This process, one of the core mechanisms of epigenetic regulation, plays an important role in the biological progression of OSCC. The main types of histone modifications include methylation, acetylation, ubiquitination, phosphorylation, sumoylation, and ADP-ribosylation; these modifications represent important entry points in the pathogenesis of OSCC and remain under active investigation. Although histone modifications are well characterized in OSCC tissues, their detection in saliva or plasma cfDNA/cf-chromatin remains exploratory and requires further methodological development.

##### Biomarkers of Histone Acetylation

Histone acetylation is a reversible process that adds acetyl groups to lysine residues of histones, catalyzed by histone acetyltransferases (HATs). Histone deacetylases (HDACs) can remove acetyl groups to restore the positive charge of histones, enhance histone-DNA binding, promote chromatin condensation, and ultimately inhibit transcriptional activity. The balance between histone acetylation and deacetylation is essential for proper gene expression regulation and is involved in many biological processes, including cell growth and development, DNA repair, responses to environmental stimuli, and tumor progression. Most tumor cells exhibit global histone hypoacetylation.

Evidence indicates specific changes in the levels of two histone deacetylases, namely HDAC1 [116] and HDAC2 [117], in OSCC. The mRNA and protein levels of HDAC2 in OSCC and precancerous tissues were significantly elevated relative to those in normal mucosa (*p* < 0.001). HDAC2 overexpression was negatively correlated with tumor histological differentiation (r = −0.63) and positively correlated with advanced TNM stage (III/IV vs. I/II, *p* = 0.008), suggesting a key role in the malignant transformation of OSCC. HDAC2 enhances HIF-1α protein stability through deacetylation modification, activates hypoxia-induced signaling pathways, and significantly promotes OSCC invasion and migration [118]. HDAC1 overexpression in OSCC can transcriptionally inhibit miR-154-5p, relieve its inhibitory effect on proliferating cell nuclear antigen (PCNA) [119], enhance DNA replication efficiency, accelerate the cell cycle, and drive tumor proliferation.

##### Biomarkers of Histone Methylation

Histone hypomethylation plays an important role in accelerating OSCC tumorigenesis. Histone methylation can dynamically regulate the methylation status of lysine and arginine residues on histone H3/H4, precisely mediate chromatin remodeling, activate or inhibit the expression of target genes, and determine cell fate. Some studies have shown regulatory network dysregulation in HNSCC. Decreased H3K36me levels are observed in approximately 20% of HNSCC cases, which is mainly attributed to a loss-of-function mutation in the histone methyltransferase NSD1 and a missense mutation (lysine to methionine) at histone H3K36M. Additionally, loss of H3K36me decreases genomic stability and accelerates malignant transformation.

Currently, histone modifications have expanded beyond the scope of classical methylation, acetylation, and ubiquitination to include phosphorylation, lactylation, succinylation, glycosylation, and β-hydroxybutyrylation. However, research in OSCC remains limited to the exploratory stage. These modifications do not act independently but rather constitute a dynamic, interacting histone code, with a pivotal role in the development of tumors by precisely regulating gene expression through spatiotemporally specific combinations.

Future research is necessary to employ multi-omics integration, high-throughput profiling, and functional validation to systematically elucidate the regulatory network governing histone modifications in OSCC and advance the translational potential of these insights for precision oncology.

#### 4.2.4. Biomarkers of Non-Coding RNA

As key regulators of the epigenome, non-coding RNAs (ncRNAs) primarily modulate gene expression via RNA methylation, histone modification, and chromatin remodeling. They participate in multiple biological processes, such as development, cell proliferation, and differentiation [120], and are associated with diseases such as tumors [121]. MicroRNAs (miRNAs) and long non-coding RNAs (lncRNAs) constitute two major classes of ncRNAs.

##### miRNA

MicroRNAs are approximately 20–24 nucleotides in length and regulate gene expression at the post-transcriptional level by targeting the 3’UTR of mRNA. In OSCC, miRNA expression profiles showed characteristic dysregulation, further regulating the malignant phenotypes of tumor cells, including proliferation, apoptosis, invasion, metastasis, and drug resistance. The role of miRNAs as important diagnostic markers in OLK has attracted considerable attention [122,123,124,125,126].

Among the miRNAs—miR-21, miR-486-5p, miR-24-3p, and miR-1307-5p—function as oncogenic drivers, promoting proliferation, invasion, or metabolic reprogramming by targeting tumor suppressor genes or regulating cell cycle pathways. Conversely, miR-31, miR-181b, and Let-7a exhibit tumor-suppressive roles, with Let-7a specifically inhibiting aerobic glycolysis via ADAMTS9-AS2-mediated binding to HK2. Several miRNAs, including miR-146a and miR-185, regulate OSCC progression by modulating autophagy, angiogenesis, and broader signaling networks. Notably, miR-424 promotes epithelial-to-mesenchymal transition in tongue SCC, while miR-375 recruits PRC2 complexes to silence tumor suppressor genes. However, the mechanisms underlying miR-125b and miR-184 regulation remain unclear or tissue-specific, underscoring the need for further research. Table 2 summarizes the core functional mechanisms and implications of key miRNA and circRNA biomarkers in OSCC [127,128,129,130]. 

Among these, miR-21, miR-31, miR-181b, Let-7a, miR-146a, miR-185, miR-486-5p, miR-10b-5p, and miR-24-3p have been validated in small-sample, single-center studies, with expression patterns varying by biospecimen type (tissue, plasma, serum, or saliva). For instance, Let-7a shows downregulation in blood but upregulation in saliva, while miR-146a exhibits the opposite trend between tissues and saliva, highlighting the importance of specimen-specific interpretation. In contrast, several markers, including miR-1307-5p, miR-375, miR-424, miR-125b, miR-184, and the circular RNAs circPRMT5 and circRNF13, remain at the exploratory stage and require further validation in larger cohorts. Notably, the majority of these candidates are involved in disease progression and prognosis, whereas only a subset (e.g., miR-21, Let-7a, miR-185) exhibits potential for diagnostic application. These findings underscore the growing promise of non-coding RNAs as liquid biopsy biomarkers, while also revealing the critical gap between discovery and clinical implementation [130,131,132,133,134,135].

The inconsistency in miR-31 expression patterns across studies is mainly due to the inherent challenges of comparing data under heterogeneous experimental conditions. Specifically, differences in data normalization and quantification strategies, variability in cohort size and composition, and heterogeneity in patient clinical history and enrollment criteria may have profound effects on outcomes. Beyond methodological aspects, biologic factors may also contribute to this divergence. In saliva, miRNA stability may be influenced by its localization in extracellular vesicles, which can protect it from enzymatic degradation. Thus, differences in the relative abundance of extracellular vesicles in the analyzed samples may affect the detectable expression levels of specific miRNAs, including miR-31 and miR-146a (Table 2 and Table 3).

##### LncRNA

Long non-coding RNAs (lncRNAs) are key regulators of many biological processes. They are characterized by more than 200 nucleotides in length and a lack of protein-coding capacity, representing a distinct layer of regulation in gene expression. The abnormal expression of lncRNAs is associated with the initiation, progression, and treatment resistance of tumors [133]; however, the function of most lncRNAs has not been elucidated. lncRNAs act as competing endogenous RNAs (ceRNAs) to bind miRNAs (miRNA sponge effect) or as structural scaffolds for chromatin modification complexes to guide epigenetic remodeling, which is one of the main mechanisms with key carcinogenic functions in OSCC [134,135].

LncRNAs exhibit superior stability and higher relative abundance in biofluids, including plasma, serum, and saliva, compared to other RNA species. These properties render them highly promising candidates to develop non-invasive liquid biopsy biomarkers for the prognostic assessment of OSCC. Although circulating lncRNA biomarkers have not yet been translated into routine clinical practice for OSCC, robust clinical trial evidence demonstrates their exceptional diagnostic sensitivity and specificity in disease management. Among them, *HOTAIR*, *MALAT1*, *PVT1*, and other lncRNAs are significantly overexpressed in OSCC tissues; therefore, they are gradually emerging as new diagnostic markers and therapeutic targets by interfering with tumor suppressor signaling pathways (such as the HOTAIR/miR-7/EGFR axis) and maintaining cancer stemness characteristics. *LncOCMRL1* is overexpressed in OSCC metastatic tumor tissues and cells. Functional studies have shown that *lncOCMRL1* overexpression promotes the growth and metastasis of OSCC cells in vivo and in vitro. Mechanistically, *lncOCMRL1* can induce epithelial-mesenchymal transition (EMT) by inhibiting *RRM2* ubiquitination, thereby promoting OSCC cells’ proliferation, invasion, and migration [136].

In studies investigating lncRNAs as liquid biopsy markers in peripheral blood (plasma/serum), 15 lncRNAs were involved; the vast majority (13) were based on plasma samples, while only *AC007271.3* and *LOC284454* were based on serum samples. Among the lncRNAs with clearly reported expression changes, most showed an upregulation trend (e.g., *PAPAS*, *NCK1-AS1*, and *GAS5*), whereas *CASC2* showed decreased expression in recurrent patients. Regarding diagnostic efficacy, the combination of four lncRNAs (*ENST00000412740*, *NR_131012*, *ENST00000588803*, and *NR_038323*) and the combination strategy of *AC007271.3* with *TSGF* and *SCCA* showed strong clinical application potential. Additionally, lncRNAs such as *HOXA11-AS*, *LINC00964*, *MALAT1*, and *LOC284454* have been proposed as potential circulating biomarkers; however, their specific expression changes and diagnostic efficacy remain to be further clarified. Overall, plasma lncRNAs show broad potential for early diagnosis, stage differentiation, prognosis assessment, and treatment monitoring of OSCC, but existing studies still face challenges such as limited sample sizes, lack of standardized procedures, and insufficient multi-center validation (Table 4).

Several studies have confirmed the detectable presence of the lncRNAs *HOTAIR* and *MALAT1* in salivary samples from patients with OSCC. Notably, *HOTAIR* expression is significantly elevated in the saliva of OSCC patients with lymph node metastasis, suggesting it as a potential biomarker for a rapid, non-invasive diagnostic approach for OSCC management via liquid biopsy approaches [151]. However, a key limitation of this study is its relatively small sample size. In a separate investigation, Shieh et al. examined circulating lncRNA *XIST* levels in saliva and reported that reduced or absent *XIST* expression is significantly associated with an increased risk of OSCC [152] (Table 4).

##### CircRNAs

Circular RNAs (circRNAs) are a class of covalently closed circular RNA molecules that are resistant to RNA exonuclease degradation and are more stable than linear RNAs. Therefore, circRNAs offer unique advantages as a biomarker for disease diagnosis and prognosis. circRNAs are expressed with tissue and cell specificity and play an important regulatory role in various physiological and pathological processes.

In OSCC, circRNAs promote tumor progression through the following mechanisms: First, circRNAs can act as microRNA sponges, specifically binding miRNAs [152]. For example, circDOCK1 adsorbs miR-1297 to upregulate HOXA9, and circGDI2 adsorbs miR-146a-5p to activate ADAM10. Second, circRNAs can mediate protein interactions: circTP53 binds USP10 to stabilize the p53 protein [153], and circSNX5 interacts with STAU1 to regulate splicing [139]. Third, circRNAs regulate signaling pathways through diverse mechanisms; for example, circHIPK3 activates the PI3K/AKT pathway [154], and circANKS1B promotes metastasis through TGF-β signaling. Previous studies have elucidated the mechanism by which HPV promotes immune escape in HNSCC via circe7-driven epigenetic modifications, suggesting a potential immunotherapy strategy for HNSCC, combining anti-PD-1 and anti-TIM-3 inhibitors. Moreover, methylation modifications in circular RNAs have increasingly been recognized as promising candidate biomarkers. However, they are currently still at the stage of laboratory research, awaiting strict clinical verification. Its value in liquid biopsy should be further explored.The core innovation of DNA methylation markers lies in the application of a “dual regulation” mode. The synergistic characteristics of “global hypomethylation and local hypermethylation” in OSCC enable multi-gene combination detection that not only covers overall tumor burden (e.g., LINE-1) but also accurately identifies key driver genes (e.g., p16 and TGM3), thereby significantly increasing specificity. The breakthrough of non-coding RNA markers is reflected in the application of exosome carriers. Salivary exosomal miRNAs and circRNAs are far more stable than free nucleic acids and can reflect the state of the tumor microenvironment, providing highly sensitive tools for early screening [155].

Although histone and RNA modifications are rarely used in clinical practice, their value lies in establishing a “diagnosis-treatment” linkage. m^6^A modification-related IGF2BP1 inhibitors are expected to become targeted therapeutic agents for OSCC metastasis. Nucleosome mapping, as an emerging approach, offers the advantage of reflecting overall chromatin structural abnormalities. Future studies should integrate DNA methylation and non-coding RNA markers to develop a more comprehensive epigenetic diagnostic model.

## 5. Discussion

### 5.1. Clinical Application of Liquid Biomarkers for OSCC and Remaining Gaps

The evidence supporting the use of liquid biomarkers for the diagnosis of primary OSCC is evolving from the initial discovery sample set to the stage of multi-center validation and diagnostic equipment assessment. A large number of proteomics and transcriptomics studies have shown that multi-indicator detection combinations can achieve clinically significant accuracy; for example, one study reported a detection combination containing four proteins, with a sensitivity of 87.5% and a specificity of 80.5% [156]. Additionally, studies evaluating the CD44/total protein ratio in oral rinse fluids have confirmed the feasibility of longitudinal monitoring and early detection in high-risk populations [157].

Although oral liquid biopsy has made these advancements, there are still significant gaps in its true clinical application. Among them, the main challenge lies in the diversity of research designs. Many diagnostic device trials and observational reports have different endpoint indicators, sampling procedures, and analysis methods, which severely limit comparisons between different studies [158,159]. Although standardized detection methods such as enzyme-linked immunosorbent assay (ELISA) and qPCR have good repeatability, exploratory platforms-especially liquid chromatography-mass spectrometry (LC-MS) and non-targeted metabolomics-will have reduced reliability unless strict centralized quality control is implemented [160]. Moreover, patient-to-patient differences caused by factors such as oral hygiene, smoking, inflammation, diet, and microbiota are major confounding factors, which reduce specificity in the general population. Therefore, many promising biomarkers perform best only in high-risk high-abundance cohorts [161].

Furthermore, comprehensive public data on the diagnostic performance of body fluids and multicenter reproducibility is still limited. To achieve wider clinical application, large-scale, multicenter randomized trials and prospective validation studies guided by unified standard operating procedures (SOPs) are needed.

Currently, although numerous saliva biomarkers have been discovered, This is largely due to the short half-life of most circulating biomarkers in blood, and the fact that the majority of promising candidates—such as miR-21 [162], miR-1307-5p, miR-185, miR-486-5p, miR-10b-5p, and miR-184 [163]—are encapsulated in exosomes, which, despite their high accuracy and tissue specificity (e.g., miR-184), still face significant barriers to routine clinical translation [164].

### 5.2. Conceptual Framework for the Clinical Management of OSCC Using Epigenetic Liquid Biopsy

Based on the principle of full-cycle management, a clinical translation pathway for epigenetic liquid biopsy in OSCC can be established and integrated with detection platforms suitable for different stages, thereby forming a closed-loop precision oncology management system. Grounded in unmet clinical needs, this system systematically delineates the diagnostic and therapeutic continuum into five sequential, clinically defined stages (C1 through C5) and aligns each stage with temporally appropriate epigenetic biomarkers and analytically optimized detection modalities.

For risk stratification and screening: Given the non-invasive nature and operational convenience of saliva sampling, saliva qPCR represents a robust, cost-effective platform for rapid epigenetic profiling in high-risk populations—particularly long-term smokers and heavy drinkers. Candidate biomarkers for such analysis include miR-21 and miR-375 expression levels, as well as LINE-1 methylation status.

For early diagnosis and differential assessment: For individuals with positive screening results from high-risk groups or those with suspected lesions in clinical settings, more sensitive tests are required—such as next-generation sequencing based on bisulfite conversion or digital methylation-specific PCR—to quantify the methylation of multiple loci of circulating tumor DNA (e.g., p16, MGMT) or exosomal circRNAs in saliva or plasma.

For prognostic stratification and treatment response monitoring: Plasma-based droplet digital PCR (ddPCR) or methylation-specific PCR (MSP) enables quantitative assessment of molecular biomarkers, such as *HDAC2* mRNA expression or *HOXA5* [165,166] promoter hypermethylation, to guide risk-adapted clinical decision-making.

For MRD detection following curative-intent surgery: This represents a critical clinical intervention for mitigating recurrence risk. Utilization of an ultra-deep, targeted methylation sequencing panel—covering established biomarker loci such as *SEPT9*—enables sensitive detection of ctDNA methylation signatures at trace concentrations in plasma. This approach holds significant promise for ultra-early MRD identification and subsequent, timely refinement of postoperative therapeutic strategies.

For longitudinal monitoring of recurrence: A comprehensive approach can be adopted for regular multi-modal monitoring, including targeted methylation sequencing and plasma analysis combined with histone modification analysis (e.g., H3K27ac ChIP-qPCR).

**Figure 3:**
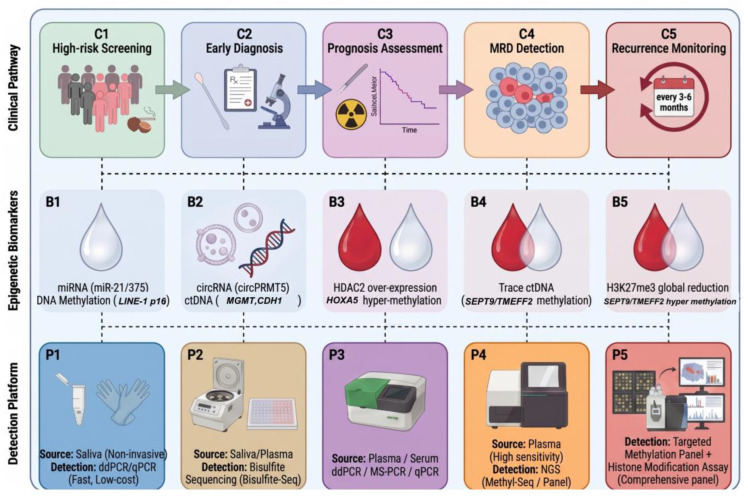
Proposed conceptual framework for integrating epigenetic liquid biopsy into OSCC clinical management. Note: This framework represents a theoretical model proposed by the authors based on current literature, rather than an established clinical guideline.

This framework represents a theoretical model proposed by the authors based on current literature, rather than an established clinical guideline. This dynamic longitudinal strategy will facilitate comprehensive closed-loop management—encompassing initial disease risk stratification and early detection, objective assessment of therapeutic efficacy, and proactive intervention to mitigate recurrence—thereby enabling end-to-end disease control (Figure 3).

### 5.3. Core Detection Platforms: Scenario-Driven Technological Adaptation

The comprehensive and full-cycle management of liquid biopsy applications needs to be realized based on a hierarchical detection platform architecture. Each platform’s technical specifications determine its optimal application method in clinical settings.

Real-time quantitative PCR (qPCR) and Rapid Detection Technologies: qPCR offers well-established methodology, operational simplicity, cost-effectiveness, and rapid turnaround time (typically within hours), making it highly suitable for frontline screening of single or limited biomarkers. Emerging rapid detection modalities, particularly nanomaterial-enhanced biosensors, demonstrate promising improvements in analytical sensitivity following further optimization and represent a pivotal advancement toward robust, scalable point-of-care testing solutions. Although qPCR offers operational simplicity and cost-effectiveness, its clinical utility in OSCC liquid biopsy is constrained by limited sensitivity, an inability to detect low-abundance ctDNA in early-stage disease or to provide multi-omics information or fragment size analysis. Furthermore, conventional qPCR provides only binary or semi-quantitative readouts, lacking the resolution required for precise monitoring of dynamic epigenetic changes. Consequently, while suitable for frontline screening of high-prevalence targets, qPCR is increasingly being supplemented or replaced by digital PCR and next-generation sequencing for comprehensive epigenetic profiling.

Next-generation sequencing (NGS) and Methylation-Specific Sequencing: NGS, including WGBS and reduced-representation bisulfite sequencing, enables unbiased, base-resolution profiling of genome-wide methylation patterns. These approaches facilitate systematic identification of differentially methylated regions (DMRs), integrative multi-omics mapping (e.g., coupling methylation with miRNA and lncRNA profiles), and de novo molecular classification. Although constrained by higher per-sample costs and computationally intensive bioinformatic analysis, NGS remains indispensable for hypothesis-generating discovery, biomarker validation, and precision prognostication. However, at present, it is only suitable for large-scale data research in the laboratory.

Droplet Digital PCR (ddPCR): ddPCR achieves absolute quantification without reliance on standard curves by partitioning samples into tens of thousands to millions of nanoliter-scale droplets, each serving as an independent amplification microreactor. This confers exceptional sensitivity (detection limit < 0.1% mutant allele fraction)—critical attributes for post-treatment MRD surveillance and longitudinal tracking of trace-level ctDNA biomarkers. This technology can accurately measure a wide range from extremely low to significantly high concentrations, even in the presence of potential interference from various substances in saliva. In the clinical setting, ddPCR has undergone extensive multi-center validation in various types of cancers. Specifically, in HPV-positive head and neck cancer, the sensitivity of ddPCR in detecting ctDNA in saliva and plasma is approximately 70%, while that of qPCR is only 20.6%. This significant difference supports the use of ddPCR for monitoring minimal residual disease.

Electrochemical Field-Effect Transistor-Based Immobilized Reaction Monitoring (EFIRM): EFIRM is an innovative electrochemical biosensing platform that enables rapid, non-invasive, and direct detection of molecular biomarkers from biological fluids—such as saliva and plasma—without requiring nucleic acid extraction, purification, or enzymatic amplification steps. In oncology diagnostics, EFIRM has been rigorously validated for sensitive and specific detection of clinically relevant somatic mutations, including EGFR exon 19 deletions and L858R substitutions, in both saliva and plasma samples. The platform achieves single-nucleotide variant (SNV) detection within 30 min using minimal sample volumes (e.g., <50 µL), thereby supporting high-efficiency liquid biopsy applications. Its clinical utility was first established through the non-invasive identification of EGFR mutations in ctDNA isolated from saliva of patients with non-small cell lung cancer. Further clinical investigations have demonstrated EFIRM’s robust performance in longitudinal monitoring of therapeutic response and early prediction of disease recurrence—capabilities that consistently outperform conventional methodologies, including qPCR and NGS [167].

## 6. Challenges and Future Prospects

**Figure 4:**
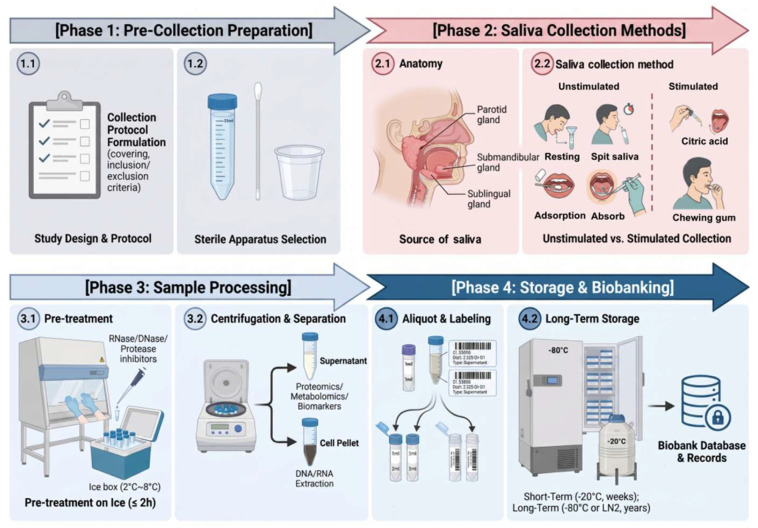
Standardized Protocol for the Collection of Human Saliva Samples. The images were made with the assistance of NanoBanana 2.

The field of oral liquid biopsy still faces many challenges. First, compared to blood samples, the collection of saliva samples requires further standardization in clinical research applications to improve the comparability and reproducibility of subsequent data (Figure 4). Moreover, the oral cavity is in direct contact with the external environment, making the situation more complex. Saliva secretion is easily influenced by external factors, necessitating further optimization through standardized collection protocols. Additionally, the information obtained by liquid biopsy cannot fully represent the complete tumor profile; it remains influenced by tumor burden and heterogeneity and lacks the morphological characteristics of tissue cells at the histopathological level. Therefore, it is currently necessary to combine liquid biopsy with histopathological examination to cover the entire process of OSCC clinical management. Finally, due to the high-dimensional characteristics of genomic data and the large patient population affected by OSCC, liquid biopsy technology faces demands for large-scale clinical implementation. The clinical technical costs are substantial, and the regulatory pathway remains unclear, which greatly limits its clinical application. Moreover, there is currently a lack of multicenter, large-scale prospective clinical validation, and most liquid biomarkers are still in the research stage and cannot yet be applied to clinical practice.

Future directions include: (1) promoting multi-omics integration and AI empowerment to build a comprehensive predictive model; (2) developing low-cost and portable detection technologies to improve accessibility; (3) expanding to “diagnosis-treatment” integration, such as targeted therapies based on epigenetics (such as histone deacetylase inhibitors); and (4) determining clinical efficacy through large cohort studies and promoting regulatory science to accelerate product transformation.

## 7. Conclusions

In conclusion, the application of epigenetic biomarkers in liquid biopsy for OSCC remains in its nascent stage, with ongoing efforts focused on analytical validation and clinical translation. DNA methylation markers exhibit a dual pattern of global hypomethylation and locus-specific hypermethylation; however, current evidence is predominantly derived from small-scale, single-center studies, and robust, multicenter clinical validation is still lacking. RNA modification markers—particularly those assessed at the tissue or cellular level—have not yet demonstrated consistent feasibility for detection in biofluids, and their clinical utility in liquid biopsy settings requires further substantiation. Histone modification markers have not been reliably detected in saliva or plasma to date; existing assays remain experimental, and significant improvements in assay sensitivity, specificity, and reproducibility are needed. Among ncRNAs, miRNAs have been repeatedly identified across multiple biofluids—including serum, plasma, and saliva—and show promise for early detection and prognostic stratification; nevertheless, inter-study heterogeneity in expression profiles, methodological variability, and insufficient cohort sizes continue to impede clinical adoption. LncRNAs demonstrate encouraging performance in diagnosis, tumor staging, and post-treatment recurrence monitoring, yet none have advanced to routine clinical implementation. CircRNAs represent the most exploratory category, with limited published data and an urgent need for well-designed clinical studies to establish analytical validity and clinical relevance. Collectively, while epigenetic markers hold substantial promise for enhancing liquid biopsy-based management of OSCC, successful translation into clinical practice hinges upon addressing critical challenges—including standardized validation frameworks, analytically robust and scalable detection platforms, and adequately powered prospective trials.

## Figures and Tables

**Table 1:** Biomarkers of DNA Methylation.

Represent Genes	Aberrant Methylation Types	Sample Verification Stage	Central Role in OSCC	References
***CCND1***	Hypomethylation	Tissue markers of prognostic Saliva samples have not yet undergone testing	It encodes Cyclin D1, drives the G1/S phase transition and promotes abnormal cell proliferation.	[53,54]
***EGFR***	Hypomethylation	Tissue markers of progression Saliva samples have not yet undergone testing Therapeutic target	It encodes epidermal growth factor receptor, enhances proliferation signals, and is associated with treatment resistance.	[55]
***AIM2***	Hypomethylation	Tissue markers of diagnostic Potential therapeutic target	It promotes the activation of inflammasomes and creates a microenvironment of chronic inflammation and immunosuppression.	[56,57,58,59,60,61,62]
***CEACAM1***	Hypomethylation	Cellular experiment verification	It regulates cell differentiation, proliferation and apoptosis, degrades the matrix barrier and enhances angiogenesis, and promotes tumor invasion and metastasis.	[63,64,65]
***LINE-1***	Hypomethylation	Tissue markers of progressive and prognostic Saliva samples have not yet undergone testing	Pan-cancer biomarkers related to disease recurrence and overall survival rate.	[66,67,68,69,70,71,72]
***PI3KCA***	Hypomethylation	Tissue markers of progressive The relationship with methylation needs to be verified	A key signaling hub for cell growth, survival, and metabolism that influences other epigenetic modifications.	[73,74,75]
***SURVIVIN***	Hypomethylation	Tissue markers of progressive Saliva samples have not yet undergone testing	Enhancing mitotic activity and driving abnormal cell proliferation; Inhibiting programmed cell death and maintaining tumor cell survival.	[76,77]
***EZH2***	Hypomethylation	Tissue markers of progressive Saliva samples have not yet undergone testing	Hypomethylation.	[78]
***KCNC4***	Hypomethylation	Tumor tissue/Cell	Functions of K intercellular transport and cell membrane depolarization.	[79]
***MAGE-A***	Hypomethylation	Tissue markers of diagnostic Saliva samples have not yet undergone testing	The developed model had 100% positive and 83.5% negative predictive values.	[80]
***p16/CDKN2A***	Hypermethylation	Tissue markers of diagnostic	Key negative regulators of the cell cycle, whose inactivation leads to uncontrolled cell cycle. DNA methylation represents the primary epigenetic mechanism responsible for transcriptional silencing.	[81,82,83,84,85,86]
***MGMT***	Hypermethylation	Tissue markers of diagnostic and prognostic Saliva samples have not yet undergone testing	DNA repair enzymes, which accumulate genomic damage after silencing, are associated with lymph node metastasis and poor prognosis.	[87,88,89,90]
***TGM3***	Hypermethylation	Tissue markers of diagnostic Saliva samples have not yet undergone testing Potential therapeutic target	Transglutaminase, loss of expression was associated with high risk of cancerization and poor prognosis of OLK.	[91]
***CDH1***	Hypermethylation	Tissue markers of diagnostic and prognostic Saliva samples have not yet undergone testing	It encodes E-cadherin, whose silencing leads to loss of cell adhesion and promotes invasion and metastasis.	[92,93,94,95]
***DAPK1***	Hypermethylation	Cellular experiment verification	Pro-apoptotic kinases, silencing allows cancer cells to evade programmed death.	[96,97]
***TFPI2***	Hypermethylation	Tissue markers of diagnostic Saliva samples have not yet undergone testing	Blocking the activity of various enzymes involved in biological processes, to be further confirmed.	[98,99,100,101,102]
***SOX17***	Hypermethylation	Cellular experiment verification	The hypermethylation of the *SOX17* gene promoter can further promote the abnormal activation of the Wnt signaling pathway in OSCC. There is a significant correlation between poor CCRT response and low survival rate.	[103,104,105,106]
***PTEN***	Hypermethylation	Cellular experiment verification	It occurs in the stage of potentially malignant oral diseases.	[107]
***TSPYL5***	Hypermethylation	Cellular experiment verification	It was negatively correlated with the degree of differentiation of OSCC.	[107]
***SFRP1***	Hypermethylation	Cellular experiment verification	Inhibition of Wnt pathway function.	[92]
***HOXA5***	Hypermethylation	Tissue markers of diagnostic and prognostic Saliva samples have not yet undergone testing	As a diagnostic and prognostic biomarker.	[108,109]

**Table 2:** miRNA and circRNA biomarkers.

Biotype	miRNA	Expression Changes	Suggested Clinical Implications	Research Stage
Tissues plasma and saliva	**miR-21**	Upregulation	Diagnosis progression and prognosis	Small sample single-center validation
	**miR-31**	Upregulation or Downregulation	Progression and prognosis	Small sample single-center validation
	**miR-181b**	Upregulation	Diagnosis and prognosis	Small sample single-center validation
	**Let-7a**	Downregulation in blood and Upregulation in saliva	Diagnosis progression and prognosis	Small sample single-center validation
	**miR-146a**	Downregulation in tissues and Upregulation in saliva	Progression and prognosis	Small sample single-center validation
	**miR-185**	Downregulation in the serum, plasma, and saliva	Diagnosis progression and prognosis	Small sample single-center validation
	**miR-486-5p**	Upregulation in the serum, plasma, and saliva	Progression and prognosis	Small sample single-center validation
	**miR-10b-5p**	Downregulation in the serum, plasma, and saliva	Progression and prognosis	Small sample single-center validation
	**miR-24-3p**	Upregulation in the serum, plasma, and saliva	Progression and prognosis	Small sample single-center validation
	**miR-1307-5p**	Upregulation in the serum, plasma, and saliva	Progression and prognosis	Experimental exploration stage
Tissues and cell	**miR-375**	Downregulation	Progression and prognosis	Experimental exploration stage
	**miR-424**	Upregulation	Progression and prognosis	Experimental exploration stage
	**miR-125b**	Downregulation	Progression and prognosis	Experimental exploration stage
	**miR-184**	Downregulation	Progression and prognosis	Experimental exploration stage
Tissues and cell	**circPRMT5**	Upregulation	Progression and prognosis	Experimental exploration stage
	**circRNF13**	Upregulation	Progression and prognosis	Experimental exploration stage

**Table 3:** The Core functional mechanisms/implications biomarkers of miRNA and circRNA.

Types of Non-Coding RNA	Representative Molecule	Core Functional Mechanisms/Implications
**miRNA**	**miR-21**	Cancer-promoting miRNA, targeting tumor suppressor genes, promotes proliferation and invasion.
	**miR-31**	Tumor suppressor miRNA, whose loss of expression promotes tumor progression.
	**miR-181b**	It acts as a tumor suppressor at tumor initiation.
	**miR-424**	Targeting TGFBR3 to promote epithelial-to-mesenchymal transition and migration of tongue squamous cell carcinoma cells requires more studies to clarify the specific role of miR-424 in oral cancer.
	**miR-375**	Recruit PRC2 complexes to silence tumor suppressor genes or act as ceRNAs that promote metastasis.
	**miR-125b**	The mechanism is not yet clear.
	**miR-184**	The expression is tissue-specific.
	**Let-7a**	ADAMTS9-AS2 drives the binding of let-7a-5p to HK2, thereby inhibiting cell growth in OSCC by eliminating aerobic glycolysis.
	**miR-146a**	Regulate the progression of OSCC through multiple mechanisms.
	**miR-185**	Inhibition of OSCC progression through autophagy, angiogenesis and other mechanisms.
	**miR-486-5p**	Significant upregulation was observed in stage II OSCC. As each stage of OSCC progresses, the upregulation increases by 3 to 4 times.
	**miR-10b-5p**	Compared with the control group, a 1.5 to 2 times decrease was observed.
	**miR-24-3p**	By regulating the cell cycle regulatory genes, the proliferation of OSCC was promoted.
	**miR-1307-5p**	By inhibiting the cancer-related genes THOP1, EHF, RNF4, GET4 and RNF114, to promote OSCC.
**circRNA**	**circPRMT5**	As a ceRNA of miR-203a-3p, it upregulates E2F3 and promotes proliferation.
	**circRNF13**	It binds and stabilizes IGF2BP1, enhances ITGB1 expression, and drives metastasis.

**Table 4:** Summary of IncRNA biomarkers for liquid biopsy of OSCC.

Biotype	lncRNA	Expression Changes	Suggested Clinical Implications	References
Plasma	*CCAT2*	Recurrent patients: decrease Non-recurrent patients: increase	Low expression is associated with local recurrence; overexpression is related to increased cell proliferation and may affect prognosis	[136]
	*PAPAS*	OSCC: upregulation	It can distinguish stage I OSCC from healthy controls; high plasma levels are associated with poorer overall survival	[137]
	*NCK1-AS1*	OSCC: upregulation	It can differentiate oral ulcers from early OSCC; it has value for early diagnosis and prognosis	[138]
	*GAS5*	Progression of OSCC patients: upregulation	It is significantly correlated with treatment response; it is upregulated in patients with disease progression (poor prognosis)	[139,140,141]
	*HOTAIR*	OSCC and lymph node metastasis patients: upregulation	It can be used for prognosis detection	[142,143]
	*ENST00000412740*	Differential expression (microarray screening)	It can be used for early diagnosis and staging, with good diagnostic efficacy when combined with ENST00000588803 NR_131012 and NR_038323combined.	[144]
	*NR_131012*	Differential expression (microarray screening)	It can be used for early diagnosis and staging, with ENST00000412740 ENST00000588803 and NR_038323 combined	[145]
	*ENST00000588803*	Differential expression (microarray screening)	It has good diagnostic efficacy when combined with ENST00000412740, NR_131012, and NR_038323 combined	[145]
	*NR_038323*	Differential expression (microarray screening)	It has good diagnostic efficacy when combined with ENST00000412740 and NR_131012 ENST00000588803	[145]
	*MALAT1*	Upregulated in tumor patients and those with lymph node metastasis patients	It may be used for prognosis detection	[145]
Serum	*AC007271.3*	It has not been clarified yet.	Combined with TSGF and SCCA, it can serve as a potential circulating molecular marker for OSCC diagnosis, suggesting that OSCC-specific lncRNAs can be released into the circulation	[146,147]
	*LOC284454*	It has not been clarified yet.	It may serve as a serum biomarker for clinical management of OSCC	[148]
Saliva	*MAL* *A* *T1*	upregulation	Prognosis	[149]
	*XIST*	upregulation	Prognosis	[150]

## Data Availability

No new data were created or analyzed in this study. Data Availability Statement is not appliable.

## Abbreviations

**Abbreviation**	**Full name**
OPMDs	Oral Potentially Malignant Disorders
OSCC	Oral Squamous-Cell Carcinoma
HNSCC	Head and Neck Squamous-Cell Carcinoma
OED	Oral epithelial dysplasia
OLK	Oral Leukoplakia
CTCs	Circulating tumor cells
ctDNA	Circulating tumor DNA
cfDNA	Cell-free DNA
DMRs	Differentially methylated regions
HPV	Human papillomavirus
PCR	Quantitative Polymerase chain reaction
OS	Overall survival
MRD	Minimal residual disease
